# Candida lusitaniae, an Emerging Opportunistic Pathogen in Immunocompetent Populations: A Case Report

**DOI:** 10.7759/cureus.43211

**Published:** 2023-08-09

**Authors:** Muhammad Haseeb ul Rasool, Gowri Swaminathan, Asma U Hosna, Salman Ishfaq, Theo Trandafirescu

**Affiliations:** 1 Medicine, Icahn School of Medicine at Mount Sinai, Queens Hospital Center, New York, USA; 2 Internal Medicine, Icahn School of Medicine at Mount Sinai, Queens Hospital Center, New York, USA

**Keywords:** immunocompetent, fungal infection, opportunistic infections, empyema, candida lusitaniae

## Abstract

*Candida lusitaniae* is an emerging opportunistic pathogen in immunocompromised hosts and hospitalized patients. However, the incidence is low in immunocompetent hosts. Because of their characteristic similarities, *C. lusitaniae* may be confused with other fungal species, such as* Candida tropicalis*,* Candida parapsilosis*, and even *Saccharomyces cerevisiae*. Recently reported cases of serious infections caused by *C. lusitaniae* have proven detrimental, and some cases reported amphotericin resistance. Here, we present a case report of empyema caused by *C. lusitaniae* in an immunocompetent patient who was admitted to the intensive care unit and intubated for acute hypoxic respiratory failure. This case demonstrates the importance of recognizing this organism and initiating early treatment for the prevention of fatal complications.

## Introduction

*Candida* species are a part of normal flora, which are most commonly found in the human gastrointestinal tract, respiratory tract, skin, genital tract, and oral cavity. Invasive candidiasis is one of the most common causes of morbidity and mortality in immunocompromised patients. Patients who undergo bone marrow transplants and are admitted to the intensive care unit (ICU) are at high risk for invasive candidiasis [1]. *Candida* infections are the second most common cause of catheter-related infections. *Candida albicans* is associated with high morbidity and mortality issues, but it is not the only species associated with human infections. *Candida lusitaniae* is an opportunistic pathogen that has been reported recently in immunocompromised hosts [2]. It is considered a least pathologic emerging pathogen that is susceptible to conventional antifungal therapies, but* C. lusitaniae* has been reported to be resistant to fluconazole and amphotericin B [2]. Among all cases of *Candida* infections, *C. lusitaniae* has been reported to be responsible for 19.3% of cases of fungemia in immunocompromised patients and 1.7% of cases of candidiasis in the general population [3]. Besides fungemia and urinary tract infections (UTI), *C. luisitaniae* can cause meningitis, empyema, and peritonitis.

Here, we present a case of *C. lusitaniae* empyema in an immunocompetent patient who was admitted to the ICU for sepsis due to pneumonia complicated by empyema.

## Case presentation

A 54-year-old male with past medical history of hypertension and alcohol use disorder presented to the emergency room with dull right flank and back pain for one week, associated with worsening dyspnea. The back pain started a week before the presentation while he was moving boxes at home. He denied any associated numbness, weakness, paresthesia, urinary retention, constipation, or fecal incontinence. The patient had gradually worsening dyspnea that especially worsened with the onset of back pain, was experiencing orthopnea, and had difficulty taking deep breaths. These were associated with the swelling of his ankle, partly associated with the recent start of amlodipine for hypertension. He denied any recent paroxysmal nocturnal dyspnea or worsening exercise tolerance. Regarding past social history, he was a social smoker in his teenage years but has not smoked since the age of 20 years. He also denied any family history of chronic diseases, including cancers.

On examination, he was found to be uncomfortable, diaphoretic, and in obvious shortness of breath. Other than a respiratory rate of 30 breaths per minute, his vital signs were within normal limits. Pulmonary exam revealed bilateral crackles, which were worse on the right than left. He was using accessory muscles and had a stony dullness on the percussion on the right side. There was bilateral pitting edema on bilateral extremities extending up to the knees. Laboratory results revealed a white cell count of 27.92 x 10^3^/ml and a lactate of 2.4 mmol/L. Chest X-ray and subsequent CT chest revealed a large right-sided pleural effusion with atelectasis of the right lung. Bedside point-of-care ultrasound revealed loculated pleural effusion with multiple small-size pockets, none of which were drainable. He was started on broad-spectrum intravenous (IV) antibiotics and was started on bilevel positive airway pressure (BiPAP) worsening hypoxia.

On the following day, interventional radiology (IR) successfully placed a chest tube, which drained 1400 ml of clear pleural fluid. The chest tube was connected to suction via the Argyle™ Thora-Seal™ III chest drainage unit (Cardinal Health Inc., USA). After a successful drainage of pleural effusion, he was transitioned from BiPAP to a high-flow nasal cannula requiring 50 L/min oxygen to saturate up to 93%. Pleural fluid analysis revealed an exudative effusion with lactate dehydrogenase (LDH) of 800 U/L, and a gram stain was found to be negative. Hypoxia was attributed to micro-atelectasis due to ongoing pneumonia and extrinsic compression from parapneumonic effusion. He was started on bronchodilators and incentive spirometry. The output of the chest tube decreased with symptomatic improvement in the shortness of breath. However, repeat imaging showed loculated pleural effusion for which intrapleural thrombolysis was performed with dornase and alteplase, resulting in a drainage of 2000 ml of pleural fluid with an improvement in oxygen requirements. 

**Figure 1:**
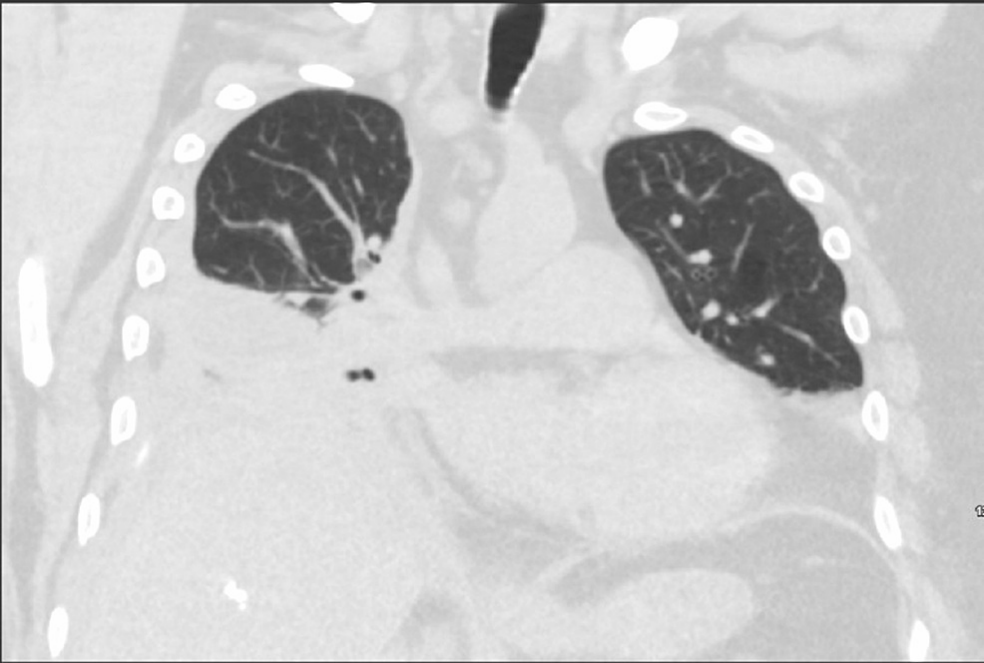
CT chest demonstrating right-sided pleural effusion

However, on the subsequent day, he started to complain of worsening pain at the site of the chest tube insertion and developed worsening dyspnea, for which he was transitioned to BiPAP. On laboratory results, the white cell count was worsening, so the antibiotic spectrum was broadened to vancomycin and meropenem. Interval CT chest revealed improved pleural effusion but worsened infiltrates (Figure 1). The white cell count improved with the change of antibiotics and with thrombolysis therapy. Due to ongoing renal injury, he was started on IV furosemide. However, due to his altered mental status, inability to protect the airway, and worsening oxygen requirements on BiPAP, he was intubated and started on mechanical ventilation. Renal function was non-responsive to trials of diuretics, so he was started on continuous renal replacement therapy (CRRT).

**Figure 2:**
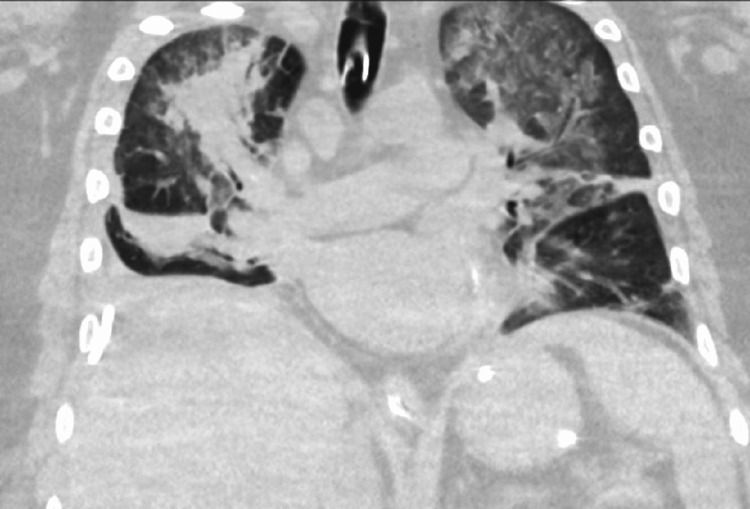
Interval CT chest demonstrating worsening of pulmonary infiltrates

He continued to be febrile despite being on meropenem, as vancomycin had to be held for acute renal injury. Due to new onset infiltrates on the CT chest, he was started on levofloxacin (Figure 2). He eventually required pressor support for hypotension. Urine culture from a prior clean catch sample resulted positive for *Candida*, for which he was started on caspofungin based on local antibiogram and infectious disease recommendation. His hemodynamics improved, so he was transitioned from CRRT to regular hemodialysis. Infectious disease is recommended to rule out tuberculosis, the further workup for which was negative. Immunoglobulin E (IgE), c3/c4, perinuclear anti-neutrophil cytoplasmic antibodies (p-ANCA), cytoplasmic nti-neutrophil cytoplasmic antibodies (c-ANCA), HIV, hepatitis B and C serology, *Legionella,* and GenMark were negative. Due to significant eosinophilia, he was started on doxycycline and stress dose steroids, resulting in improvement in the white cell count and hemodynamic measures. Urine culture from the urine collected at the time of admission and pleural fluid cultures from the pleural fluid collected at time of chest tube insertion resulted in *C. lusitaniae*. Because of positive cultures at two distant sites, he was labeled to have disseminated candidiasis. The antibiotic spectrum was narrowed to meropenem and caspofungin.

**Figure 3:**
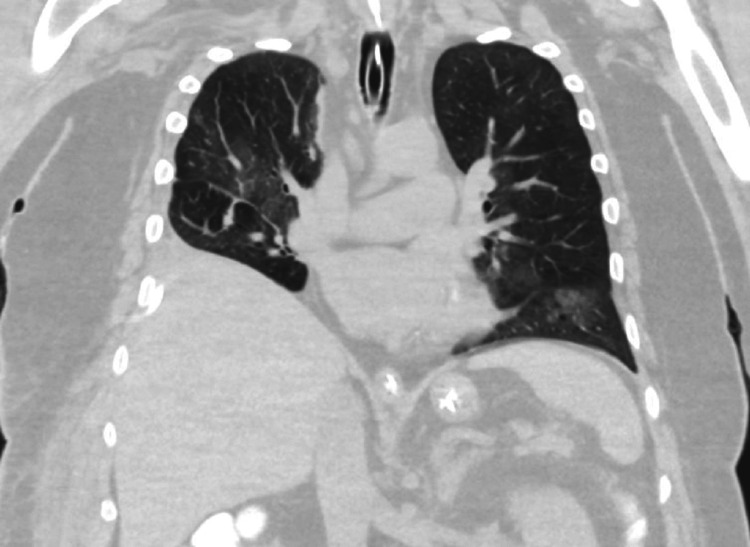
CT chest revealing resolution of the pneumonia

The patient was followed with interval cultures to monitor the resolution of the infection. As soon as the patient was afebrile for 24 hours, the dialysis catheter was removed and replaced after 24 hours after collecting the blood cultures. Due to the minimal chest tube output, the chest tube was removed after a trial of clamping for 24 hours. As there is no established guideline on the duration of antifungal treatment in *C. lusitaniae* sepsis, per infectious disease recommendations, the patient was continued on caspofungin and followed up with daily urine cultures, until three consecutive cultures were negative. After the negative cultures were achieved, a permcath was placed for continued hemodialysis. With improving clinical status, pressor support was progressively weaned off, and he was extubated successfully and eventually transferred to the medical floors for continued care. Repeat CT chest revealed the resolution of infiltrates (Figure 3). Oxygen requirement progressively weaned off, and he was able to maintain saturation on room air. With the recovery of renal function, dialysis was stopped, and the permcath was removed. Due to prolonged ICU stay, he developed critical care illness and required two-person support to walk. He was discharged to a subacute rehabilitation facility for physical therapy before he could be discharged to home in self-care. He was found to be performing well and had a stable renal function at the subsequent follow-up in the next six months.

## Discussion

Fungal infections in immunocompromised individuals have been on the rise and have posed quite a challenge to the medical fraternity. This increase in incidence seems to be multifactorial, with an increase in the number of immunocompromised individuals and the rising resistance to antifungal agents [2]. *Candida* infections have predominantly been found in individuals in the backdrop of prolonged broad-spectrum antibiotic use, immunosuppressive therapy use, blood or solid organ transplants, cancer, diabetes mellitus, or AIDS [4].

The poster child *C. albicans*, a common commensal organism, is known to cause the most opportunistic infections. The lesser-known species* C. glabrata*,* C. krusei*,* C. parapsilosis*, *C. dublieniesis*,* C. tropicalis*, *C. kefyr*, and *C. guilliermondii* are the most frequently isolated species within this eclectic group, while *C. inconspicua*,* C. lusitaniae*,* C. norvegensis*, and *C. rugosa* are found much less frequently [5]. These species lack the classic virulence factors possessed by *C. albicans* in the form of the hyphal phenotype and the ability to adhere to the oral epithelium and high proteinase excretion and therefore cause less severe infections [4]. Among the infections caused by *Candida* spp., *C. lusitaniae* is responsible for 19.3% of fungemia in cancer patients and 1.7% of all cases of genitourinary candidiasis in ambulatory patients [2]. The incidence of *C. lusitaniae* in immunocompetent individuals has not been well explored; therefore, our 54-year-old immunocompetent patient with disseminated candidiasis with *C. lusitaniae* is a unique addition to the existing literature on fungal infections. 

The clinical data on candidemia caused by *C. lusitaniae* is few and far between. In a retrospective analysis conducted at MD Anderson Advanced Cancer Center in Houston, Texas, between 1988 and 1999, 12 cases of* C. lusitaniae* candidemia were identified, of which seven were breakthrough infections (58%; four on amphotericin B and three on fluconazole). Eight out of the 12 patients had hematologic malignancies or were post-hematopoietic cell transplant, and most patients (75%) were granulocytopenic at the time of infection [6]. A subsequent study in the same institution from 1998 to 2013 revealed *C. lusitaniae* isolates in 19 of 1,395 *Candida* bloodstream infections. Most of the patients had hematologic malignancies, and seven out of 19 were breakthrough infections. We did not find any evidence of hematological conditions that could predispose our patient to having an invasive infection from* C. lusitaniae* [7].

While it is widely known that disseminated fungal infections are mostly seen in immunosuppressed individuals, our case throws light on how some species of *Candida* can escape immune detection in an immunocompetent host. *C. guilliermondii*, the phylogenetically closest species to *C. lusitaniae*, has been explored enough to understand this very interaction. Murine phagocytic cells, bone marrow cells, and spleen cells have a better ability to kill* C. guilliermondii* when compared with human monocytes [8,9]. *C. guilliermondii* cells trigger much higher levels of cytokines, such as tumor necrosis factor-alpha (TNF-α), interleukin-6 (IL-6), IL-1β, and IL-10 in contrast to *C. albicans* [10]. Due to its close phylogenetic relationship to *C. guilliermondii*, it could be surmised that a similar immune phenomenon was the cause for the widespread damage by *C. lusitaniae* in our presented case. In 2012, Silva et al. reported in a retrospective study of 62 patients with pulmonary fungal infections at Coimbra University Hospital, Portugal, that there is a similar prevalence of this kind of infection in both immunocompetent and immunocompromised patients [11].

*C. lusitaniae* is an extremely uncommon opportunistic infection that causes invasive infections in immunocompromised patients with underlying malignancy and/or those undergoing chemotherapy [6,12,13]. While our patient did not present with the classical risk factors for *C. lusitaniae*, we surmise that the use of broad-spectrum antibiotics early in the patient’s hospital course may have increased his susceptibility to the fungal infection with *C. lusitaniae*; it has been documented as contributive to colonization and infection in patients with prolonged hospitalizations and use of broad-spectrum antibiotics [14].

*C. lusitaniae* is different from other clinically significant *Candida* species as it can quickly develop in vivo resistance to amphotericin B upon exposure to the agent, through high-frequency phenotypic switching from susceptibility to resistance associated with distinct morphologies [15]. Along with the rapid mutation rate, the ability to form biofilms makes *C. lusitaniae* infection challenging to treat [16], through overstimulation of drug efflux pumps along with limitation of the diffusion of the antifungal drugs through biofilms [17]. As for our patient, we initiated the antifungal regimen with caspofungin only after a trial with broad-spectrum antibiotics, such as piperacillin-tazobactam, vancomycin, and meropenem. We chose an echinocandin as the antifungal agent of choice in this patient because of his ongoing renal impairment and its better pharmacologic profile when compared to the liposomal amphotericin B. A 16-day course of caspofungin was necessary for our patient to achieve consistently *Candida*-free cultures.

## Conclusions

*C. lusitaniae*, a haploid yeast, is a rare etiology of disseminated candidiasis in the immunocompromised population and even more so in immunocompetent individuals. *C. lusitaniae* is highly prone to developing resistance against amphotericin B and azoles; therefore, the initial antifungal therapy of choice should be an echinocandin. The duration and decision to continue or hold treatment should be guided by the clinical response, both in terms of subjective parameters, such as symptoms, and objective measures, such as vital signs, serial microbiology workup, and other site-specific investigations. To the authors’ knowledge, there are no similar cases reported in the literature so far.
